# The senescent niche hypothesis: microglial dysfunction and replacement strategies in drug-resistant epilepsy

**DOI:** 10.3389/fimmu.2026.1807871

**Published:** 2026-04-10

**Authors:** Jingheng Wu, Miaomiao Li, Yetong Shi, Shuai Wang

**Affiliations:** 1 Department of Functional Neurosurgery of Shengjing Hospital of China Medical University, Shenyang, Liaoning, China; 2 Tianjin Key Laboratory of Cerebral Blood Flow Reconstruction and Head and Neck Tumor New Technology Translation, Tianjin Neurosurgical Institute, Tianjin Huanhu Hospital, Tianjin, China; 3 Huanhu Hospital Affiliated to Tianjin Medical University, Tianjin Medical University, Tianjin, China; 4 Liaoning Clinical Medical Research Center in Nervous System Disease, Shenyang, Liaoning, China

**Keywords:** blood–brain barrier, epilepsy, ferroptosis, iron–senescence axis, microglial senescence, microglial replacement therapy, senolytics

## Abstract

Epilepsy is one of the most prevalent neurological disorders, affecting over 70 million individuals worldwide. However, despite the introduction of more than 30 anti-seizure medications over three decades, approximately 30% of patients continue to suffer from drug-resistant epilepsy (DRE). Here, we advance the “Senescent Niche Hypothesis,” proposing that the epileptogenic focus in DRE harbors a pathological accumulation of senescent microglia that have lost homeostatic surveillance capacity and acquired a toxic secretory phenotype. We present the “Iron–Senescence Axis” as the mechanistic driver: recurrent seizure-induced blood–brain barrier disruption leads to chronic parenchymal iron deposition; microglia accumulate iron through erythrophagocytosis and sustain sub-lethal ferroptotic stress—characterized by lipid peroxidation, mitochondrial dysfunction, and DNA damage—that drives their irreversible transition to a senescent state rather than acute cell death. Once senescent, these microglia paradoxically acquire resistance to ferroptosis through lysosomal iron sequestration, occupy the niche indefinitely, and perpetuate epileptogenesis via the senescence-associated secretory phenotype (SASP), establishing a positive feedback loop. Converging transcriptomic and experimental evidence from both human surgical specimens and rodent models substantiates this framework, demonstrating that senolytic clearance of senescent cells significantly reduces seizure burden and can prevent epilepsy development. Building on these findings, we evaluate two complementary therapeutic strategies: senolytic therapy using dasatinib plus quercetin (D+Q) for selective elimination of senescent cells, and the Microglial Intervention Strategy for Therapy and Enhancement by Replacement (MISTER) for comprehensive niche reconstitution through CSF1R inhibitor-mediated microglial depletion followed by donor cell engraftment. We critically assess donor cell sources, advances in non-genotoxic conditioning, and CSF1R-inhibitor resistant donor cells that may enable clinical translation. This synthesis argues that targeting the senescent microglial niche may represent a disease-modifying approach that shifts the therapeutic focus from seizure suppression to neuroimmune niche restoration.

## 1 Introduction

Epilepsy encompasses a group of neurological disorders defined by an enduring predisposition to generate epileptic seizures, with neurobiological, cognitive, and psychosocial consequences (1). Affecting over 70 million individuals worldwide, it remains a leading cause of neurological disability. Seizures are classified by the International League Against Epilepsy (ILAE) according to onset type (focal, generalized, or unknown), and epilepsy syndromes span a spectrum from self-limited childhood conditions to severe, lifelong encephalopathies. Central to brain homeostasis are microglia, the resident myeloid cells of the central nervous system (CNS), which derive from yolk sac erythromyeloid progenitors during embryogenesis and are maintained throughout life by colony-stimulating factor 1 receptor (CSF1R) signaling (2). In the healthy brain, microglia perform continuous surveillance of the parenchymal microenvironment via dynamic process extension, mediate synaptic pruning during development and plasticity, clear cellular debris and apoptotic cells, and regulate neuronal activity through purinergic signaling (3). This homeostatic repertoire positions microglia as critical gatekeepers of neural circuit integrity—and, when compromised, as potential contributors to disease progression.

### 1.1 Drug-resistant epilepsy: the limitations of neurocentric approaches

Over the past three decades, regulatory agencies have approved more than 30 newer anti-seizure medications (ASMs) spanning diverse molecular targets—from voltage-gated sodium channel modulators (lacosamide, cenobamate) and synaptic vesicle protein ligands (levetiracetam, brivaracetam) to AMPA receptor antagonists (perampanel) (4). Despite the development of numerous new drugs, the rate of treatment failure remains unchanged: approximately one-third of patients continue to experience DRE, defined by the International League Against Epilepsy (ILAE) as failure to achieve sustained seizure freedom after adequate trials of two appropriately chosen and tolerated ASM regimens (5, 6).

This therapeutic plateau is particularly intractable in temporal lobe epilepsy with hippocampal sclerosis (TLE-HS), the most common surgically remediable epilepsy syndrome. Anterior temporal lobectomy achieves seizure freedom in 60–80% of well-selected candidates (7, 8), yet a substantial proportion of patients remain ineligible for resection owing to bilateral or poorly localized seizure onset, and others experience late recurrence years after initially successful surgery (9). For these individuals, no disease-modifying intervention exists (10).

These limitations suggest that alternative therapeutic strategies should be considered. The overwhelming majority of existing ASMs operate within a neurocentric paradigm: they target ion channels, neurotransmitter receptors, or synaptic machinery to suppress the electrical manifestations of seizures (11). While effective for symptom control in pharmacoresponsive phenotypes, these agents function as “anti-seizure” rather than “anti-epileptogenic” drugs—they dampen hyperexcitable network output without addressing the diseased microenvironment that sustains seizure susceptibility (11). We suggest that the persistent drug resistance rate despite three decades of ASM development may indicate that the epileptogenic substrate extends beyond neuronal excitability to include the neuroimmune microenvironment of the epileptogenic focus.

### 1.2 From neuroinflammation to immunosenescence: the evolving understanding of microglial dysfunction

Non-neuronal contributions to epileptogenesis have traditionally been conceptualized through the framework of neuroinflammation. Under this paradigm, microglia—the resident myeloid cells of the central nervous system (CNS)—were viewed primarily as pathological effectors that, upon “activation,” release pro-convulsant cytokines (IL-1β, TNF-α, HMGB1) capable of lowering seizure thresholds by modulating glutamatergic and GABAergic neurotransmission (12, 13). This framework motivated clinical trials repurposing anti-inflammatory agents—corticosteroids, minocycline, IL-1 receptor antagonists (anakinra), and caspase-1 inhibitors (VX-765)—for DRE, with largely disappointing results (14).

We propose that these therapeutic failures may reflect an incomplete understanding of the microglial state in chronic epilepsy. Emerging transcriptomic evidence and critical reappraisals have challenged the M1/M2 polarization dichotomy (15), revealing that microglia in established DRE may undergo a transition toward cellular senescence—a state characterized by irreversible homeostatic failure rather than sustained inflammatory activation (16, 17). Cellular senescence, as codified by international consensus criteria (18), is characterized by stable cell-cycle arrest mediated by cyclin-dependent kinase inhibitors p16INK4a and p21CIP1, activation of the DNA damage response (DDR) with γH2AX foci, nuclear envelope deterioration with loss of Lamin B1, lysosomal expansion detectable as senescence-associated β-galactosidase (SA-β-gal) activity, and—critically—acquisition of the senescence-associated secretory phenotype (SASP), a complex paracrine program comprising pro-inflammatory cytokines, chemokines, growth factors, and matrix metalloproteinases that actively remodels the tissue microenvironment (18, 19).

This distinction has important therapeutic implications. Anti-inflammatory strategies aim to modulate the output of activated cells, but they cannot reverse cellular senescence. The failure of anti-inflammatory trials in DRE is thus not surprising if the predominant microglial state is senescence rather than activation—a state that is not effectively targeted by conventional anti-inflammatory pharmacology.

### 1.3 Convergent evidence: cellular senescence as a therapeutic target in epilepsy

Recent studies have provided converging evidence that cellular senescence contributes to epileptogenesis and may represent a therapeutic target in DRE (**Table 1**; for a comprehensive list of molecular senescence markers, see **Supplementary Table 1**). In the pilocarpine-induced TLE mouse model, Khan et al. (21) demonstrated that senescent cells—predominantly microglia—accumulate in the hippocampus within two weeks of status epilepticus, preceding spontaneous seizure onset. Importantly, senolytic clearance of these cells reduced seizure burden and, in a subset of animals, prevented epilepsy development (detailed in Section 3.1.2). Ge et al. (20) used Patch-seq on neurons from 36 brain samples of patients with DRE and identified a senescent subpopulation of cortical pyramidal neurons (elevated p21, p53, γH2AX; diminished Lamin B1; increased SA-β-gal), demonstrating that neuronal senescence occurs in developmental epilepsy lesions (FCD). In contrast, Khan et al. (21) showed that microglial senescence predominates in TLE. Ribierre et al. (22) showed that senolytic treatment with dasatinib plus quercetin (D+Q) reduced senescent cell burden and seizure frequency in an mTOR-related epilepsy model (Section 3.1.2). Together, these findings indicate that cellular senescence actively contributes to network hyperexcitability and is amenable to pharmacological intervention.

**Table 1:** Evidence summary for cellular senescence in drug-resistant epilepsy.

Study	Year	Species/tissue	Key findings	Senescence markers	Predominant cell type
Ge et al. (20)	2025	Human (resected DRE tissue: FCD, TLE)	Patch-seq identified senescent pyramidal neuron subpopulation; 5-fold increase in senescent cells vs controls	p21↑, p53↑, COX-2↑, γH2AX↑, SA-β-gal↑, Lamin B1↓, CCL2↑, NFKBIA↑	Neurons (pyramidal)
Khan et al. (21)	2025	Human (TLE) + Mouse (pilocarpine SE)	Senescent cells accumulate within 2 weeks post-SE; D+Q reduces seizures by 50%, prevents epilepsy in 1/3	p16↑, p21↑, SA-β-gal↑	Microglia (predominantly)
Ribierre et al. (22)	2024	Human (FCDII) + Mouse (Mtor^S2215F^)	Dysmorphic neurons exhibit senescence; D+Q reduces seizure frequency	p16↑, p53↑, SA-β-gal↑, SASP expression	Neurons (dysmorphic)
Shahidehpour et al. (17)	2021	Human (aging, AD, LBD, LATE)	Dystrophic microglia associated with disease, not healthy aging; iron homeostasis changes	Ferritin light chain↑, morphological dystrophy	Microglia
Streit et al. (16)	2009, 2020	Human (AD, aging)	Dystrophic microglia precede tau pathology; proposed as senescence phenotype	Morphological changes, ferritin↑	Microglia

Summary of key studies providing evidence for cellular senescence in drug-resistant epilepsy and related conditions. Studies are organized by publication year and include information on species/tissue examined, principal findings, senescence markers identified, and the predominant senescent cell type. The evidence suggests that neuronal senescence predominates in developmental lesions (FCD/mTORopathies), while glial (particularly microglial) senescence predominates in acquired epilepsies (TLE-HS). Abbreviations: DRE, drug-resistant epilepsy; FCD, focal cortical dysplasia; TLE, temporal lobe epilepsy; SE, status epilepticus; D+Q, dasatinib plus quercetin; AD, Alzheimer’s disease; LBD, Lewy body disease; LATE, limbic-predominant age-related TDP-43 encephalopathy; SASP, senescence-associated secretory phenotype.

↑ denotes upregulation/increased expression; ↓ denotes downregulation/decreased expression.

### 1.4 The senescent niche hypothesis: scope and objectives

Building on this evidence, we advance the “Senescent Niche Hypothesis”: the epileptogenic focus in DRE constitutes a pathological microenvironment—an immunosenescent niche—dominated by dysfunctional senescent microglia that cannot be rehabilitated by conventional pharmacological means.

**Figure 1:**
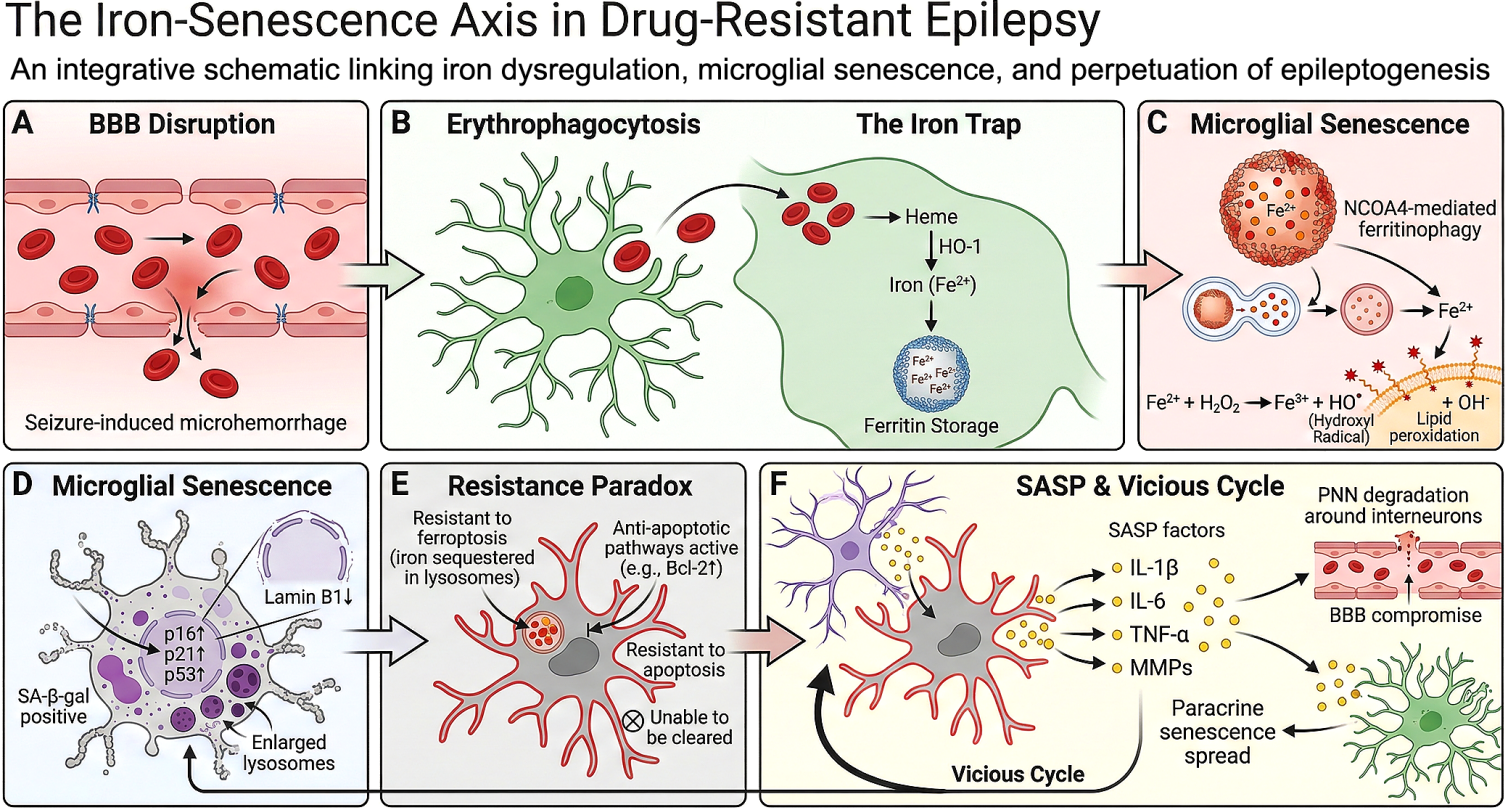
The iron–senescence axis: an integrative schematic linking iron dysregulation, microglial senescence, and perpetuation of epileptogenesis in drug-resistant epilepsy. **(A)** Recurrent seizures induce microhemorrhages through BBB disruption, resulting in extravasation of erythrocytes into the brain parenchyma. **(B)** Microglia engulf extravasated erythrocytes via erythrophagocytosis and metabolize heme through heme oxygenase-1 (HO-1), releasing ferrous iron (Fe^2+^) for storage in ferritin. **(C)** Under chronic iron overload (“The Iron Trap”), NCOA4-mediated ferritinophagy releases excess Fe^2+^ from saturated ferritin into the cytoplasm, where Fenton chemistry (Fe^2+^ + H_2_O_2_ → Fe^3+^ + HO• + OH^-^) generates hydroxyl radicals that drive lipid peroxidation—the biochemical hallmark of sub-lethal ferroptotic stress. **(D)** Accumulated oxidative damage triggers cellular senescence, characterized by upregulation of p16^INK4a^, p21, and p53, increased senescence-associated β-galactosidase (SA-β-gal) activity, loss of nuclear lamin B1, and expansion of dysfunctional lysosomes. The distinction between acute, lethal ferroptotic insult (leading to cell death) and chronic, sub-lethal ferroptotic stress (leading to senescence) determines the cellular outcome, as elaborated in Section 2.3. **(E)** The Resistance Paradox: senescent microglia become paradoxically resistant to both ferroptosis (through lysosomal iron sequestration) and apoptosis (through upregulation of anti-apoptotic pathways such as Bcl-2), rendering them unable to be cleared and creating persistent senescent cells that occupy the niche indefinitely. **(F)** Senescent microglia secrete senescence-associated secretory phenotype (SASP) factors—including IL-1β, IL-6, TNF-α, and matrix metalloproteinases (MMPs)—that degrade perineuronal nets (PNNs) surrounding interneurons, compromise BBB integrity, and induce paracrine senescence in neighboring cells, establishing a self-perpetuating vicious cycle that defines the Senescent Niche.

We propose the “Iron–Senescence Axis” as the mechanistic driver of this niche. Recurrent seizure-induced blood–brain barrier (BBB) disruption leads to chronic parenchymal iron deposition through microhemorrhage. Microglia, as the obligate phagocytes of the CNS, accumulate iron through erythrophagocytosis and experience sub-lethal ferroptotic stress—lipid peroxidation, mitochondrial damage, and DNA lesions—that activates the DDR and drives their irreversible transition to a senescent state (**Figure 1**). Once senescent, these cells paradoxically acquire resistance to ferroptotic death through lysosomal iron sequestration (the “Resistance Paradox”), occupy the niche indefinitely, and perpetuate epileptogenesis via SASP-mediated disruption of perineuronal nets, BBB integrity, and inhibitory circuit function.

**Figure 2:**
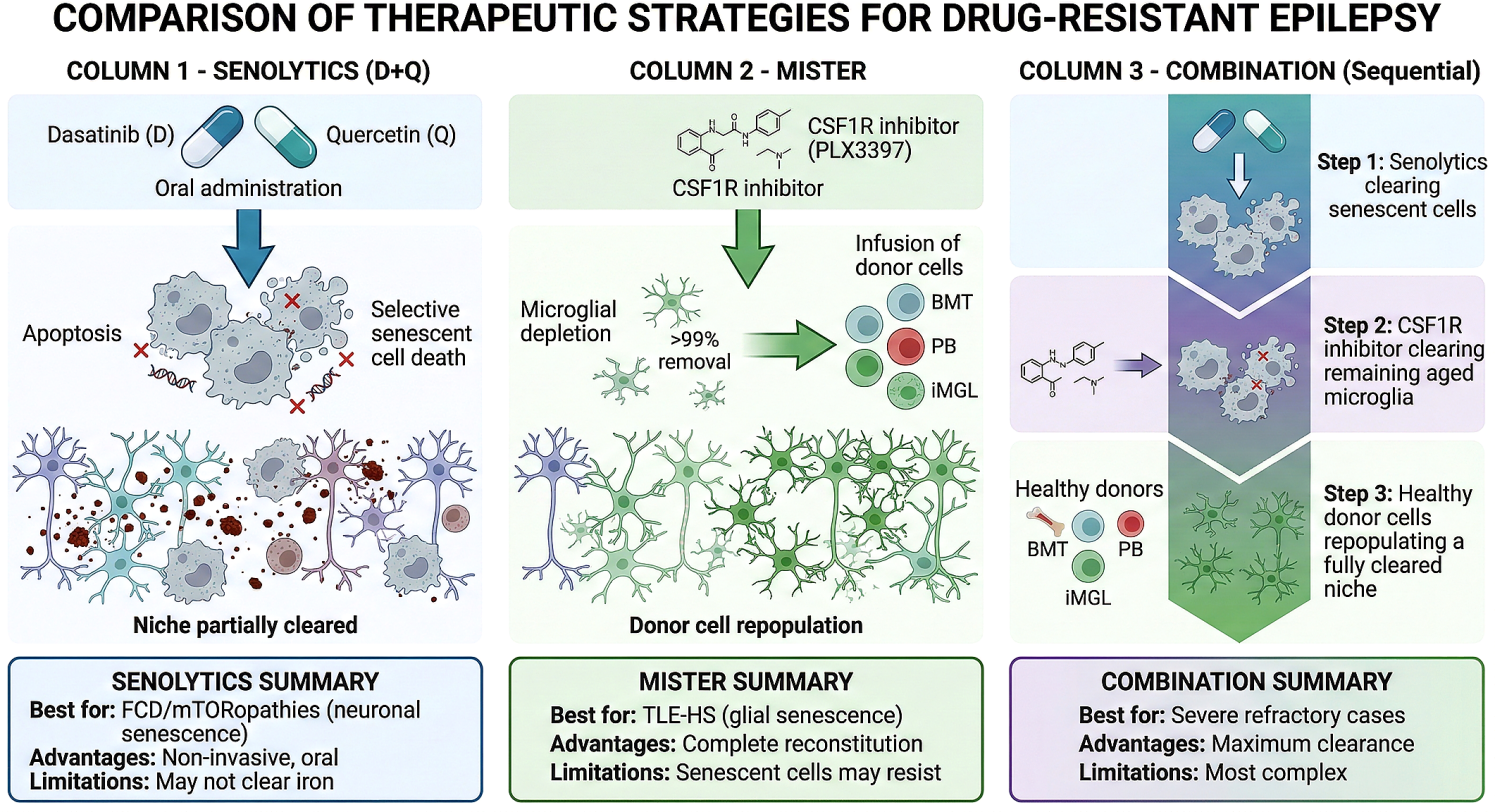
Comparison of therapeutic strategies targeting the senescent niche in drug-resistant epilepsy. Three approaches are presented in parallel columns with their respective mechanisms, advantages, and limitations. Column 1 – Senolytics (D+Q): Oral administration of dasatinib (D), a multi-kinase inhibitor, combined with quercetin (Q), a Bcl-xL inhibitor, selectively induces apoptosis in senescent cells while sparing healthy counterparts. This approach partially clears the senescent niche and is best suited for FCD/mTORopathies where neuronal senescence predominates. Advantages include non-invasive oral dosing; limitations include incomplete niche clearance and persistence of iron deposits. Column 2 – MISTER (Microglial Intervention Strategy for Therapy and Enhancement by Replacement): CSF1R inhibitor (PLX3397)-mediated depletion achieves >99% microglial removal, followed by infusion of donor cells from one of three sources: bone marrow transplant (BMT), peripheral blood (PB)-derived monocytes, or induced pluripotent stem cell-derived microglia (iMGL). Donor cells repopulate the vacated niche with healthy microglia. This strategy is best suited for TLE-HS with predominant glial senescence; however, senescent microglia may resist CSF1R inhibitor-mediated depletion (the Resistance Paradox). Column 3 – Combination (Sequential):A three-step protocol designed to overcome the limitations of either approach alone: Step 1, senolytics clear senescent cells; Step 2, CSF1R inhibitor depletes remaining aged microglia; Step 3, healthy donor cells (BMT/PB/iMGL) repopulate a fully cleared niche. This sequential three-step strategy is proposed for severe refractory cases and offers maximum niche clearance, though it represents the most complex therapeutic protocol.

This framework suggests that therapeutic approaches targeting immune niche reconstitution, rather than neuronal modulation alone, may be warranted. The subsequent sections of this review synthesize evidence for the Iron–Senescence Axis, critically evaluate two complementary strategies—senolytic therapy (D+Q) for selective elimination of senescent cells and the Microglial Intervention Strategy for Therapy and Enhancement by Replacement (MISTER) for comprehensive niche reconstitution through colony-stimulating factor 1 receptor (CSF1R) inhibitor-mediated depletion followed by donor cell engraftment (**Figure 2**)—and outline a translational roadmap for clinical implementation.

## 2 The iron–senescence axis: a metabolic pathway to microglial immunosenescence

Given the evidence that cellular senescence is present in the epileptic brain and may be amenable to senolytic intervention (Section 1.3), we now address the upstream mechanism driving microglia from homeostatic surveillance into irreversible senescence. We propose the Iron–Senescence Axis as a unifying hypothesis that links the distinctive vascular pathology of epilepsy to microglial immunosenescence and niche dysfunction (**Figure 1**). The axis proceeds through three sequential phases: hemorrhagic iron influx, microglial iron overload, and the paradoxical survival of iron-damaged cells as senescent niche occupants.

### 2.1 The hemorrhagic microenvironment: iron as the priming signal

The initiation of the senescent niche is rooted in the physical disruption of the neurovascular unit. In epilepsy, BBB dysfunction operates as both cause and consequence of seizure activity. Acute seizures induce transient BBB opening through glutamate-mediated endothelial injury, matrix metalloproteinase (MMP) activation, and pericyte dysfunction (23). Conversely, BBB leakage promotes epileptogenesis through albumin extravasation and activation of astrocytic TGF-β signaling, which impairs potassium buffering and lowers seizure thresholds (24)—establishing a bidirectional pathological loop from the earliest stages of the disease (**Figure 1A**).

Critically, BBB disruption in chronic TLE is not limited to soluble protein leakage; it is frequently accompanied by frank microhemorrhage. MRI studies employing susceptibility-weighted imaging (SWI) and T2* sequences have documented hemosiderin deposits in the hippocampi of TLE patients at rates far exceeding those in age-matched controls (25, 26), and histopathological examination of resected TLE specimens confirms hemosiderin-laden macrophages and extracellular iron deposits concentrated in regions of severe neuronal loss (26). These observations indicate that the epileptogenic hippocampus exhibits chronic iron deposition in addition to inflammation. The lysis of extravasated erythrocytes liberates hemoglobin, which degrades into free heme and catalytically active ferrous iron (Fe^2+^), creating a pro-oxidant microenvironment within the seizure focus.

### 2.2 Microglial iron accumulation: from neuroprotective scavenging to iron dyshomeostasis

Microglia are the obligate phagocytes of the CNS, uniquely positioned to clear extravasated erythrocytes through erythrophagocytosis (**Figure 1B**). Internalized red blood cells are degraded within the phagolysosomal compartment, liberating heme that is metabolized by heme oxygenase-1 (HO-1) to yield ferrous iron, carbon monoxide, and biliverdin (27). Under physiological conditions, this system is neuroprotective: liberated iron is sequestered within ferritin (comprising ferritin heavy chain 1 [FTH1] and ferritin light chain [FTL] subunits) or exported to the extracellular space via ferroportin (SLC40A1), maintaining the cytoplasmic labile iron pool (LIP) at non-toxic concentrations.

We propose that chronic, recurrent microhemorrhage in DRE may overwhelm this protective system, leading to pathological iron accumulation (**Figure 1C**). Repeated erythrophagocytosis drives progressive intracellular iron accumulation that exceeds ferritin storage capacity. Under normal conditions, ferritin-bound iron is mobilized through selective autophagy (ferritinophagy) mediated by the cargo receptor nuclear receptor coactivator 4(NCOA4) (28); however, in iron-overloaded states, even basal rates of NCOA4-mediated ferritinophagy become maladaptive: the release of iron from saturated ferritin stores exceeds the capacity for re-sequestration or export, dangerously elevating the LIP. Through Fenton chemistry (Fe^2+^ + H_2_O_2_ → Fe^3+^ + •OH + OH^-^), the resultant hydroxyl radicals preferentially attack polyunsaturated fatty acids (PUFAs) in membrane phospholipids, initiating lipid peroxidation—the biochemical hallmark of ferroptosis (29–31).

This iron-trapping mechanism has a morphological correlate in human tissue. Prior to the molecular characterization of senescence, Streit and colleagues (32, 33) systematically characterized dystrophic microglia—cells with fragmented, beaded processes, spheroid formations, and loss of finely ramified arbors—in aged human brains and Alzheimer’s disease tissue. These features, initially interpreted as degeneration or exhaustion, are now understood as morphological signatures of cellular senescence (16). Extending this work, Shahidehpour et al. (17)[**Table 1**] analyzed human post-mortem brains across multiple neurodegenerative conditions (Alzheimer’s disease, Lewy body disease, limbic-predominant age-related TDP-43 encephalopathy) and identified a critical association: dystrophic microglia were characterized not only by cytorrhexis but specifically by aberrant accumulation of ferritin light chain (FTL)—the iron-storage subunit. Crucially, this iron-loaded dystrophic phenotype was associated with disease pathology rather than healthy aging, establishing a direct link between iron dyshomeostasis and the morphological transformation of microglia to a senescent-like state. Although these observations derive from neurodegenerative rather than epileptic tissue, the mechanistic inference is compelling: if iron overload drives microglial dystrophy and ferritin accumulation in neurodegeneration, the same process is predicted to operate—and potentially with greater intensity—in the chronically hemorrhagic microenvironment of TLE-HS, where the iron burden is recurrently replenished by ongoing BBB disruption.

Complementary evidence from epileptic tissue itself supports this extrapolation. Morin-Brureau et al. (34) demonstrated that microglia in resected human TLE-HS specimens undergo morphological transformation from ramified to amoeboid phenotypes in sclerotic regions, with upregulation of activation markers (MHCII, CD68) and altered purinergic receptor profiles, suggesting a shift away from homeostatic surveillance toward a reactive state. P2RY12 is the purinergic receptor essential for microglial process motility and the capacity to sense and respond to neuronal distress (3); disruption of purinergic signaling impairs the ability of microglia to detect local network instability. This homeostatic failure—functional rather than merely morphological—is precisely the phenotype predicted by iron-driven senescence: cells that survive but can no longer perform their surveillance duties.

Recent studies have further established that microglia are among the most ferroptosis-susceptible cell types in the CNS, with iron overload triggering a distinct transcriptional program that overlaps significantly with disease-associated microglia (DAM) signatures (35, 36). We propose that the pathological state in chronic DRE involves sub-lethal ferroptotic stress rather than complete cell death: microglia experiencing chronic, low-grade lipid peroxidation and glutathione depletion survive but accumulate the cellular damage that drives senescence (37–39).

### 2.3 The “resistance paradox”: from sub-lethal ferroptotic stress to irreversible senescence

A key question arising from this framework is how microglia survive chronic iron overload: if iron overload triggers ferroptosis—an iron-dependent form of regulated cell death (29)—the question of how these microglia survive requires explanation. We propose that the answer lies in the distinction between acute, lethal ferroptotic insult and chronic, sub-lethal ferroptotic stress, and that this distinction determines whether the outcome is cell death or cellular senescence (**Figure 1D**).

#### 2.3.1 Molecular pathways linking oxidative damage to senescence

Several converging molecular pathways link sub-lethal ferroptotic stress to the senescence program. Iron-catalyzed reactive oxygen species (ROS) inflict diverse forms of DNA damage—strand breaks, base oxidation (8-oxoguanine lesions), and DNA–protein crosslinks—that activate the DNA damage response (DDR) through ATM/ATR kinases and their downstream effectors CHK1/CHK2. These kinases stabilize p53 and induce transcription of CDKN1A (p21), thereby enforcing cell-cycle arrest—the cardinal feature of senescence (40).

In parallel, iron accumulation within mitochondria disrupts electron transport chain function, exacerbating ROS production in a feed-forward loop. Damaged mitochondria that escape mitophagy release mitochondrial DNA (mtDNA) into the cytoplasm, where it activates the cGAS–STING innate immune sensing pathway, directly driving expression of SASP components—the inflammatory secretome that transforms senescent cells from passive bystanders into active agents of tissue remodeling (41). The metabolic shift toward glycolysis observed in senescent cells may itself reflect this irreversible mitochondrial damage (42). At the lysosomal level, iron accumulation—particularly as insoluble hemosiderin aggregates—impairs acidification and degradative capacity, manifesting as the expanded, dysfunctional lysosomes detected by SA-β-gal staining, the most widely used histochemical marker of senescence (43).

Thus, the iron-to-senescence transition is not mediated by a single pathway but by the simultaneous convergence of three arms of cellular damage: (i) nuclear DNA damage → DDR → p53/p21 → cell-cycle arrest; (ii) mitochondrial dysfunction → mtDNA release → cGAS–STING → SASP; and (iii) lysosomal iron loading → degradative failure → SA-β-gal positivity. Each pathway is supported by experimental evidence; their convergence in iron-overloaded microglia provides a mechanistic basis for the Iron–Senescence Axis.

#### 2.3.2 Ferroptosis resistance in senescent microglia

An additional feature of the iron–senescence relationship relevant to the pathological cycle: once microglia have entered the senescent state—a transition itself driven by iron dyshomeostasis (44) —they paradoxically become resistant to the very ferroptotic stress that induced their senescence (37) (**Figure 1E**).

Feng et al. (37) elucidated the mechanism underlying this paradox. In doxorubicin-induced senescent cells, iron accumulates preferentially in lysosomes rather than the cytoplasm. This lysosomal sequestration, mediated by reduced NCOA4-dependent ferritinophagy, effectively buffers the cytoplasmic LIP and limits the lipid peroxidation that would otherwise execute ferroptotic death. The functional significance of this compartmentalization was demonstrated experimentally: disruption of lysosomal function with chloroquine dramatically sensitized senescent cells to death, confirming that lysosomal iron retention is a critical survival mechanism rather than an incidental feature.

The therapeutic implications of this resistance paradox are profound. Senescent microglia within the epileptogenic focus are simultaneously:

- Dysfunctional—unable to perform homeostatic surveillance, synaptic pruning, or debris clearance owing to impaired purinergic signaling and disrupted lysosomal function;
- Pro-epileptogenic—actively secreting SASP factors (IL-1β, IL-6, TNF-α, MMPs, CCL2, CCL11) that degrade perineuronal nets surrounding inhibitory interneurons (45), compromise BBB tight junctions (46), and induce paracrine senescence in neighboring cells (47) (**Figure 1F**);
- We propose that senescent microglia within the epileptogenic focus may become therapy-resistant—protected from ferroptotic death by lysosomal iron sequestration and from apoptosis by upregulated senescent cell anti-apoptotic pathways (SCAPs), and potentially resistant to CSF1R inhibitor-mediated depletion, as suggested by preliminary evidence (21).

These combined features produce a ferroptosis-resistant senescent phenotype in which cells persist in the niche, potentially block repopulation by healthy microglia, and maintain disease pathology through continuous SASP secretion. The SASP itself establishes a self-reinforcing vicious cycle—senescence → SASP → BBB compromise → iron leakage → further senescence—that may explain the progressive expansion of the epileptogenic zone over time (**Figure 1F**). Critically, this mechanism provides the clearest explanation for why broad-spectrum anti-inflammatory drugs have failed in DRE: blocking individual SASP cytokines does not eliminate the senescent cells that continuously regenerate the inflammatory milieu. In this framework, the inflammatory milieu is maintained by a population of senescent cells that may need to be eliminated rather than pharmacologically modulated.

Crucially, this is not a unidirectional pathway. SASP factors secreted by senescent microglia (e.g., IL-1β, MMPs) further degrade the BBB, promoting secondary waves of iron influx. This feedback between senescence and iron influx may contribute to the progressive nature of DRE and the limited efficacy of single-target interventions.

## 3 Therapeutic strategies: from seizure suppression to immune niche reconstitution

The Iron–Senescence Axis, as elaborated in Section 2, identifies a pathological endpoint—the senescent microglial niche—that cannot be addressed by conventional anti-seizure pharmacology. If the epileptogenic focus is populated by dysfunctional, SASP-secreting, therapy-resistant senescent microglia, then therapeutic strategy must shift from modulating neuronal output to reconstituting the immune microenvironment itself. Two complementary approaches have emerged: pharmacological clearance of senescent cells through senolytics, and comprehensive niche replacement through microglial engraftment (**Figure 2**).

### 3.1 Pharmacological clearance: the senolytic approach

#### 3.1.1 Mechanism of action

Senolytics selectively induce death in senescent cells while sparing non-senescent counterparts by exploiting a fundamental vulnerability: senescent cells depend on upregulated anti-apoptotic pathways—collectively termed the senescent cell anti-apoptotic pathway (SCAP) network—for their survival (48). The most extensively studied senolytic combination, dasatinib plus quercetin (D+Q), targets complementary arms of this network. Dasatinib, a multi-kinase inhibitor originally developed for chronic myeloid leukemia, inhibits Src family kinases and ephrin receptor tyrosine kinases that are selectively upregulated in senescent cells. Quercetin, a naturally occurring flavonoid, inhibits PI3K, BCL-xL, and serpines, with preferential senolytic activity against senescent endothelial cells and adipocyte progenitors. The combination exhibits synergistic activity across diverse senescent cell types, achieving a broader efficacy spectrum than either agent alone (48).

#### 3.1.2 Preclinical evidence in epilepsy

As introduced in Section 1.3, preclinical studies have demonstrated the therapeutic potential of senolytics in epilepsy. Here we detail these findings in the context of D+Q’s mechanism of action. Ribierre et al. (22) provided the first proof-of-concept that senolytic therapy could directly modify seizure frequency in an epilepsy model: in an electroporated MtorS2215F mouse model of focal cortical dysplasia type II (FCDII), D+Q administration reduced SA-β-gal-positive cells within the electroporated cortical region and decreased spontaneous seizure frequency. Khan et al. (21) extended these findings to TLE. In the pilocarpine model, D+Q treatment initiated during the latent period produced a 50% reduction in hippocampal senescent cell burden, normalized spatial memory deficits, significantly reduced spontaneous recurrent seizures, completely prevented epilepsy development in approximately one-third of treated animals, and rescued long-term potentiation (LTP) deficits—collectively establishing senolytics as potential disease-modifying agents in epilepsy.

#### 3.1.3 Translational advantages and limitations

The principal translational advantages of D+Q include oral bioavailability, established safety profiles (dasatinib is FDA-approved for chronic myeloid leukemia; quercetin holds GRAS status), and “hit-and-run” pharmacokinetics that permit intermittent dosing rather than continuous exposure. A recent pilot study in older adults with mild cognitive impairment demonstrated that D+Q (100 mg dasatinib + 1,250 mg quercetin administered for two consecutive days every two weeks over 12 weeks) was well tolerated, with evidence of CNS penetration and preliminary signals of improved mobility and cognition (49). Ongoing work is evaluating next-generation senolytics with enhanced BBB permeability and cell-type specificity (50).

While D+Q is the gold standard senolytic combination in peripheral tissues, we acknowledge that quercetin exhibits limited BBB permeability. Future clinical translation may require nanoparticle-mediated delivery systems or the development of CNS-penetrant senolytics (e.g., small molecule Bcl-xL inhibitors targeting senescent microglia and senescent endothelial cells within the epileptogenic niche) to ensure therapeutic concentrations in the hippocampus.

In addition to senolytics, senomorphics—agents that suppress the SASP without eliminating senescent cells—merit consideration. Rapamycin suppresses SASP by inhibiting mTORC1-dependent translation of IL-1α and is particularly relevant given mTOR hyperactivation in epileptogenesis. Metformin activates AMPK and suppresses NF-κB-driven cytokine secretion, with demonstrated anti-inflammatory effects in preclinical epilepsy models. JAK inhibitors (e.g., ruxolitinib) attenuate microglial SASP through JAK/STAT blockade. However, senomorphics do not eliminate the dysfunctional, iron-loaded cells occupying the niche: they would require lifelong continuous administration and would not vacate the physical niche to allow for microglial repopulation—a prerequisite for the MISTER strategy. Senomorphics may therefore be most valuable as adjunctive agents, dampening SASP-mediated damage while senolytics or MISTER address the underlying niche pathology.

However, several limitations of senolytic monotherapy warrant consideration in the context of DRE. First, the efficacy of D+Q against senescent microglia specifically remains uncertain; the SCAP network may vary across cell types, and microglia may require different senolytic combinations than the cell types in which D+Q was originally characterized. Second, senolytic-mediated lysis of senescent cells releases their accumulated intracellular iron content into the parenchyma, potentially propagating oxidative damage and seeding new cycles of ferroptotic stress in neighboring cells. Third, the optimal therapeutic window relative to the natural history of epileptogenesis remains undefined: administration during the latent period (as in Khan et al.) may be feasible in experimental models but is difficult to translate to clinical practice, where patients typically present with established chronic epilepsy. Finally, and most fundamentally, senolytic clearance addresses the pathological occupants of the niche but does not repopulate it with functionally competent cells—leaving open the question of whether endogenous microglial repopulation can adequately restore homeostatic surveillance in a microenvironment that remains iron-loaded and structurally damaged.

These limitations motivate a more comprehensive approach: not merely clearing the senescent niche, but actively replacing it.

### 3.2 The MISTER paradigm: deplete and repopulate

#### 3.2.1 Conceptual foundation

The concept of microglial replacement therapy originated not in epilepsy but in the study of CSF1R-related leukoencephalopathy, specifically adult-onset leukoencephalopathy with axonal spheroids and pigmented glia (ALSP)—a primary genetic microgliopathy caused by heterozygous loss-of-function mutations in CSF1R (51). Because the fundamental defect in ALSP resides in microglia themselves, and homozygous CSF1R mutations can result in congenital absence of microglia (52), HSCT has been explored as a replacement strategy, with case reports documenting clinical stabilization and MRI improvement following engraftment (53). We propose that TLE-HS may share functional similarities with ALSP: although patients lack germline CSF1R mutations, their microglia may become dysfunctional due to cumulative iron overload and senescence-associated homeostatic failure, suggesting that similar replacement strategies could be explored.

We define MISTER—the Microglial Intervention Strategy for Therapy and Enhancement by Replacement—as a two-phase protocol for comprehensive immune niche reconstitution (54).

#### 3.2.2 Phase 1: niche depletion

In the depletion phase, small-molecule CSF1R inhibitors (PLX3397/pexidartinib, PLX5622/plexxikon) penetrate the BBB and induce rapid microglial apoptosis, eliminating >99% of resident microglia within 7–21 days without overt neuroinflammation or neuronal damage (55, 56). This pharmacological depletion creates a vacant niche—an open ecological space within the CNS parenchyma that is competitively available for colonization by donor-derived cells.

A critical unresolved question, however, concerns the susceptibility of senescent microglia to CSF1R inhibitor-mediated depletion. The Resistance Paradox described in Section 2.3.2 extends beyond ferroptosis resistance: emerging evidence suggests that senescent microglia may also resist CSF1R-dependent apoptosis (21), paralleling the SCAP-mediated anti-apoptotic resistance that characterizes senescent cells across tissue contexts. If confirmed, this resistance would leave senescent cells occupying niche space, actively secreting SASP factors, and potentially outcompeting incoming donor cells for limited trophic support (CSF-1, IL-34, TGF-β). Several potential solutions merit investigation: sequential senolytic–MISTER therapy combining D+Q pre-treatment to eliminate senescent cells with subsequent CSF1R inhibitor-mediated depletion of remaining non-senescent microglia; dual-target compounds that simultaneously inhibit SCAP and CSF1R signaling; and antibody–drug conjugates directed against senescence-specific surface markers, capable of delivering cytotoxic payloads to senescent cells regardless of their CSF1R dependence.

#### 3.2.3 Phase 2: niche repopulation

In the repopulation phase, donor-derived myeloid cells are introduced to colonize the vacated niche. Engrafted cells encounter CNS-specific environmental cues (TGF-β, IL-34, CSF-1) that drive transcriptional reprogramming: monocytic markers are progressively downregulated while core microglial homeostatic genes (Tmem119, P2ry12, Hexb, Sall1) are upregulated (57, 58). Whether engrafted cells fully recapitulate endogenous microglial identity remains debated and is discussed in Section 4.2.

### 3.3 Comparative analysis of donor cell sources

The clinical viability of MISTER depends critically on the selection of an appropriate donor cell source. Three principal sources are under active translational development, each with distinct advantages and trade-offs for specific clinical scenarios (**Table 2**).

**Table 2:** Comparative analysis of microglial replacement strategies.

Feature	BMD-HSCs (bone marrow)	PBDCs (peripheral blood)	iMGL (iPSC-derived)
Donor Source	Bone marrow HSCs (iliac crest aspiration)	G-CSF mobilized HSCs or monocytes (apheresis)	Reprogrammed iPSCs differentiated to microglia
Ontogeny	Definitive hematopoiesis	Definitive hematopoiesis	Primitive streak (yolk sac-like)
Collection Invasiveness	High (bone aspiration under anesthesia)	Low (blood draw/apheresis)	Low (skin biopsy or blood for iPSC generation)
Delivery Route	Intravenous (IV)	Intravenous (IV)	Intracerebral injection (IC) or IV*
Brain Engraftment Efficiency	Excellent (55-75% with conditioning)	Good (variable, depends on conditioning)	Limited (focal only without conditioning)
Long-term Survival	Permanent (self-renewing HSCs)	Variable (HSC=high; Monocyte=may require redosing)	Permanent (if successfully grafted)
Identity Fidelity	Macrophage-like initially; adapts to microglial signature over time	Macrophage-like; similar adaptation	High (transcriptionally similar to native microglia)
Gene Therapy Compatibility	Yes (ex vivo transduction)	Yes (ex vivo transduction)	Excellent (CRISPR correction before differentiation)
HLA Matching Required	Yes (allogeneic) or No (autologous)	Yes (allogeneic) or No (autologous)	No (autologous from patient iPSCs)
GvHD Risk	Present (allogeneic setting)	Present (allogeneic setting)	None (autologous)
Manufacturing Complexity	Low	Low	High (differentiation protocol, quality control)
Scalability	Good	Excellent	Limited
Regulatory Pathway	Established (HSCT precedent)	Established	Novel (cell therapy regulations)
Cost	Moderate	Low-Moderate	High
Best Clinical Application	Generalized/multifocal DRE; patients tolerating invasive procedures	Fragile patients; repeated dosing protocols; combination with CSF1Ri-resistant cells	Focal TLE/HS; precision medicine (gene-corrected); patients requiring autologous therapy

*IV delivery of iMGL with G795A CSF1R mutation combined with CSF1Ri treatment enables brain-wide engraftment.

Comparative analysis of the three principal donor sources for microglial replacement therapy. BMD-HSCs (bone marrow transplantation) offers the longest track record and excellent engraftment but requires invasive collection. PBDCs (peripheral blood) represents the most clinically practical approach with minimal invasiveness. iMGL (iPSC-derived microglia) provides the highest ontogenic fidelity and is ideal for autologous, gene-corrected applications but faces delivery challenges and high manufacturing costs. Recent advances in CSF1R-inhibitor resistant donor cells (G793A/G795A mutations) may enable peripheral delivery of iMGL with brain-wide engraftment (Chadarevian et al., 2025; Lombroso et al., 2025).

#### 3.3.1 Bone marrow-derived HSCs: the preclinical gold standard

Bone marrow-derived hematopoietic stem cells (HSCs), harvested by iliac crest aspiration under anesthesia, constitute the gold standard in preclinical MISTER studies (57). BMD-HSCs offers the highest brain engraftment efficiency documented to date—55–75% microglial replacement when combined with appropriate conditioning (59, 60)—and permanent graft survival owing to the self-renewal capacity of HSCs (**Table 2**). Donor-derived cells traffic to the CNS via the CCL2–CCR2 chemokine axis and, upon engraftment, undergo the transcriptional adaptation toward a microglial signature described above. The ontogeny of these cells is definitive hematopoiesis, distinguishing them from yolk sac-derived endogenous microglia; however, this ontogenic discordance does not preclude functional integration (57, 58).

The principal limitations of BMD-HSCs are clinical rather than biological. Collection requires invasive bone marrow aspiration under general anesthesia. In the allogeneic setting, HLA matching is required and graft-versus-host disease (GvHD) remains a substantive risk. Although autologous transplantation eliminates immunological mismatch, it does not address the underlying susceptibility of patient-derived cells to the same iron-loaded microenvironment. The established HSCT regulatory framework, however, provides a relatively clear pathway to clinical implementation. BMD-HSCs is best suited for patients with generalized or multifocal DRE who can tolerate the invasiveness of bone marrow collection and conditioning (**Table 2**).

#### 3.3.2 Peripheral blood-derived cells: the minimally invasive alternative

Peripheral blood-derived cells, obtained through G-CSF mobilization of HSCs or monocyte apheresis, provide a minimally invasive alternative that substantially lowers the procedural barrier to MISTER implementation (58). Collection requires only venipuncture or leukapheresis—a critical advantage for fragile patients or repeated dosing protocols. Delivery is intravenous, and the regulatory pathway is established based on existing peripheral blood stem cell transplantation precedent.

The trade-offs are biological rather than procedural. Brain engraftment efficiency is variable and depends heavily on the conditioning regimen employed. Long-term survival shows a bifurcation: HSC-derived engrafters demonstrate high persistence, while monocyte-derived cells may require periodic redosing to maintain therapeutic coverage (**Table 2**). Like BMD-HSCs, peripheral blood cells arise from definitive hematopoiesis and acquire a macrophage-like phenotype initially, with progressive adaptation toward a microglial transcriptional signature over time. Critically, PBDCs is the donor source most compatible with the CSF1R-inhibitor resistant cell engineering approach (described in Section 3.4), as peripheral blood cells can be readily subjected to ex vivo transduction with the G795A CSF1R variant before reinfusion (**Table 2**). Among the three donor sources, PBDCs offers the highest scalability and lowest cost, making it the most practical option for broad clinical deployment.

#### 3.3.3 iPSC-derived microglia: the precision medicine platform

Induced pluripotent stem cell-derived microglia (iMGL) represent the donor source with the highest ontogenic similarity to endogenous microglia, as iPSC differentiation protocols recapitulate primitive streak (yolk sac-like) hematopoiesis—the developmental pathway that gives rise to endogenous microglia (**Table 2**). Accordingly, iMGL achieve the highest identity fidelity of the three sources, with transcriptional profiles closely matching those of native microglia. Because iMGL can be derived from patient-specific iPSCs (generated from skin biopsy or peripheral blood), they enable fully autologous transplantation with no requirement for HLA matching and no risk of GvHD.

The unique strength of iMGL lies in their compatibility with precision gene editing. iPSCs can be subjected to CRISPR-mediated correction or enhancement at the undifferentiated stage—before microglial differentiation—allowing the generation of genetically engineered microglia tailored to the pathological environment. Potential engineering strategies include knockout of complement components (C1q, C3) to prevent aberrant synaptic pruning that contributes to inhibitory synapse loss (61); overexpression of ferritin heavy chain (FTH1) or knockdown of NCOA4 to enhance iron sequestration capacity, producing “iron-resistant” microglia pre-adapted to the hemorrhagic DRE microenvironment; and constitutive secretion of neuroprotective factors such as GDNF or adenosine (via adenosine kinase inhibition), transforming microglia from passive homeostatic cells into active therapeutic delivery vehicles (**Table 2**).

The limitations of iMGL are predominantly logistical. Manufacturing complexity is high, requiring specialized differentiation protocols and rigorous quality control. Scalability is currently limited compared with BMD-HSCs or PBDCs. Cost is the highest of the three sources. The regulatory pathway is novel, lacking the HSCT precedent that facilitates BMD-HSCs and PBDCs implementation. Most critically, iMGL currently require intracerebral injection for engraftment—a neurosurgically invasive delivery route that limits treatment to focal pathology. However, the G795A CSF1R engineering approach (Section 3.4) may overcome this barrier by enabling peripheral intravenous delivery with brain-wide engraftment in the future (59, 62). iMGL are best suited for focal TLE-HS, where precision medicine approaches and autologous sourcing are prioritized (**Table 2**).

Therefore, as summarized in **Table 2**, while BMD-HSCs offers superior durability and the most robust engraftment efficiency, iMGL represents the future of personalized medicine owing to its reduced immunogenicity, autologous sourcing potential, and unparalleled gene-editing compatibility. PBDCs, by contrast, occupies a pragmatic middle ground—offering the lowest procedural barrier and highest scalability—that may prove most suitable for initial clinical implementation and broad deployment. The limitations and safety considerations of microglial replacement therapy—including the CNS immune vulnerability window during depletion, the risk of recurrent donor cell senescence in the iron-loaded microenvironment, and the absence of MISTER studies in epilepsy models—are discussed in detail in Section 4.

### 3.4 Engineering resilience: CSF1R-inhibitor resistant donor cells

A key translational challenge for MISTER is the timing of CSF1R inhibitor treatment: the inhibitor must be administered long enough to deplete endogenous microglia, but its withdrawal permits residual host cells—potentially including senescent, SASP-secreting microglia that survived depletion—to repopulate the niche in competition with donor-derived cells. Traditional approaches required myeloablative conditioning (alkylating chemotherapy or total body irradiation) to ensure donor engraftment, an unacceptable toxicity burden for non-malignant neurological conditions.

Two converging advances have transformed this landscape. First, the development of non-genotoxic conditioning using CD45-targeted immunotoxins enables selective depletion of host hematopoietic stem cells without collateral tissue damage, facilitating donor HSC engraftment without conventional chemotherapy (63).

Second—and more transformatively for MISTER—Chadarevian et al. (62) and Lombroso et al. (59) have demonstrated that a single point mutation in CSF1R (G795A in human, G793A in mouse) confers resistance to PLX3397 and PLX5622 while preserving normal receptor signaling and microglial function. This advance addresses the timing challenge: donor cells engineered with the resistance mutation can be infused concurrently with ongoing CSF1R inhibitor treatment, allowing continuous suppression of endogenous (senescent) microglia while engrafted donor cells selectively expand into the vacated niche—providing a selective advantage to donor cells.

Chadarevian et al. (62) demonstrated that G793A knockin mice exhibit broad CSF1R inhibitor resistance, enabling efficient microglia replacement following intraparenchymal, intracisternal, or bone marrow transplantation routes. Critically, iPSC-derived human microglia engineered with the G795A mutation could also engraft via the less invasive intracisternal route, opening a path toward peripheral delivery of iMGL. In the companion study, Lombroso et al. (59) showed that peripheral intravenous delivery of G793A-expressing hematopoietic cells achieves robust brain engraftment (~55–75% microglial replacement) with minimal peripheral chimerism, while proving less toxic to neuronal and oligodendrocyte progenitors than traditional myeloablative HSCT.

The convergence of non-genotoxic conditioning, CSF1R-inhibitor resistant donor cells, and ex vivo gene editing represents a qualitative advance in the clinical tractability of microglial replacement. For DRE specifically, this platform enables a therapeutic sequence that directly addresses the pathology defined by the Iron–Senescence Axis: senolytic pre-treatment (D+Q) to debulk the senescent population; concurrent CSF1R inhibitor administration to deplete remaining non-senescent but dysfunctional microglia; and infusion of G795A-engineered donor cells—potentially carrying additional genetic modifications for iron resistance or neuroprotective factor secretion—to reconstitute a metabolically competent immune niche (**Figure 2**, Column 3).

## 4 Discussion: translational landscape and unresolved questions

### 4.1 Limitations of the current evidence

It is important to delineate what the current evidence establishes, what can be inferred, and what remains speculative. We acknowledge significant gaps in the evidence base that must be addressed before this framework can support clinical intervention.

First, no published study has yet applied MISTER specifically in epilepsy models. The therapeutic rationale presented here extrapolates from three separate literatures—ALSP microglial replacement (51–53), microglial depletion-repopulation in neurodegeneration models (54–56), and cellular senescence in epilepsy (20–22)—whose convergence remains empirically untested as an integrated intervention. Second, human neuropathological studies demonstrating senescence markers in resected DRE tissue (20, 21, 34) are inherently correlational and cannot establish causality; the presence of senescent microglia in the epileptogenic focus does not prove that they drive epileptogenesis rather than accumulate as a consequence of it. Although Khan et al.’s (21) demonstration that senolytic clearance during the latent period prevents seizure development in one-third of treated animals argues strongly for causality, replication in independent models is essential.

Third, the Iron–Senescence Axis itself, though mechanistically coherent and supported by indirect evidence from multiple disciplines, awaits direct experimental validation. Studies employing iron chelation in TLE models, genetic manipulation of microglial iron-handling genes (ferritin, ferroportin, NCOA4), or inducible microglial-specific iron overload in epilepsy-prone animals are needed to test whether iron accumulation is necessary and sufficient for microglial senescence in the epileptic brain. Fourth, mouse models imperfectly recapitulate human epilepsy and microglial biology: species differences in microglial transcriptional programs, lifespan-dependent senescence dynamics, and brain iron metabolism may limit direct translation of preclinical findings. Finally, the long-term consequences of fundamentally altering the brain’s resident immune landscape through microglial replacement remain unknown. Rigorous experiments utilizing conditional genetic models—such as inducible microglial-specific knockdown of iron-handling genes or selective ablation of senescent microglia via p16-CreERT2 driver lines—are essential to establish whether microglial senescence drives epileptogenesis or accumulates as a downstream consequence.

### 4.2 Functional equivalence over ontogenic identity in microglial replacement

A central question for MISTER concerns whether peripherally derived myeloid cells can recapitulate microglial function. Endogenous microglia derive from yolk sac erythromyeloid progenitors that colonize the brain during early embryogenesis (2), and this primitive ontogeny is reflected in a unique transcriptional identity—including expression of Sall1, Hexb, and P2ry12—that distinguishes microglia from other tissue-resident macrophage populations.

As described in Section 3.2.3, the CNS microenvironment drives substantial transcriptional reprogramming of engrafted donor cells. However, the extent of this reprogramming varies across studies. Bennett et al. (57) demonstrated that engrafted peripheral macrophages progressively upregulate core microglial homeostatic genes, whereas Cronk et al. (58) showed that peripherally derived macrophages maintain a transcriptionally distinct identity even after prolonged CNS residence. Hohsfield et al. (60) similarly found that bone marrow-derived myeloid cells resemble but do not fully replicate the endogenous microglial transcriptome after long-term brain-wide colonization.

In the context of DRE, we suggest that clinical utility may be a more relevant criterion than ontogenic identity. The endogenous microglial population in the epileptogenic focus is dysfunctional—senescent, iron-loaded, and SASP-secreting. The relevant question is whether donor-derived cells can perform the homeostatic functions that these cells have lost: synaptic surveillance, debris clearance, iron scavenging, and trophic support. Available evidence suggests that engrafted cells acquire sufficient functional competence (57, 60), and their enhanced phagocytic capacity could be specifically advantageous for clearing residual iron deposits in the DRE microenvironment. The donor source hierarchy presented in **Table 2** allows this trade-off to be calibrated to clinical need.

### 4.3 Safety considerations: from preclinical reassurance to clinical caution

The safety profile of microglial depletion and replacement requires careful evaluation. As noted in Section 3.2.2, preclinical studies demonstrate that CSF1R inhibitor-mediated microglial depletion is well tolerated in rodents (55, 56), and the clinical risks of allogeneic versus autologous donor sources are summarized in **Table 2** and Section 3.3. Here we address safety considerations that extend beyond the immediate procedural risks.

First, the depletion phase represents a window of CNS immune vulnerability. We propose that patients undergoing MISTER would require intensive ASM bridging therapy and close monitoring for both seizure exacerbation and infectious complications until donor cell engraftment is established (typically 7–14 days). The duration and severity of this vulnerability require characterization in phase I trials.

Second, the long-term consequences of microglial replacement for CNS-specific immune functions remain unknown. Whether donor-derived microglia can adequately support tumor immunosurveillance, clearance of protein aggregates, regulation of adaptive immune cell entry, and maintenance of myelin integrity across the lifespan will require extended follow-up in any clinical program. Munro et al. (64) demonstrated that microglia actively protect against age-associated brain pathologies, underscoring that long-term microglial absence carries risk—though MISTER aims for replacement rather than sustained depletion.

Third, for autologous approaches, whether patient-derived cells carry intrinsic susceptibilities—genetic or epigenetic—that might predispose them to recurrent senescence upon exposure to the DRE microenvironment is an open question that may necessitate engineered iron resistance (Section 3.3.3).

These risks must be weighed against the disease burden of refractory DRE: uncontrolled seizures, progressive cognitive decline, psychiatric comorbidity, SUDEP risk, and the irreversible tissue loss of lobectomy.

### 4.4 Critical questions requiring resolution

Several foundational questions must be addressed before MISTER can advance to clinical implementation:

Selective targeting. Whether senescent microglia can be specifically identified and eliminated *in vivo* remains to be determined. If they resist CSF1R inhibitor-mediated depletion, as the Resistance Paradox predicts (Section 2.3.2), alternative surface markers or molecular vulnerabilities that might enable their selective clearance must be identified. The identification of senescence-specific surface antigens suitable for antibody–drug conjugate targeting represents a high-priority research objective.

Therapeutic window. The optimal point in the natural history of epileptogenesis for intervention—whether during the latent period before spontaneous seizure onset, the early chronic phase, or established refractory disease—remains to be defined. Khan et al.’s (21) latent-period administration achieved the most dramatic results, but this window is rarely identifiable in clinical practice, where patients present with established epilepsy.

Phenotype durability. Whether donor-derived microglia maintain their healthy, homeostatic phenotype indefinitely within the DRE microenvironment, or eventually succumb to the same pathological pressures—iron overload, oxidative stress, and SASP-mediated paracrine senescence—that corrupted their endogenous predecessors remains an open question. If the epileptogenic microenvironment converts healthy donor cells to a senescent phenotype over time, MISTER may require periodic re-dosing or combinatorial strategies (e.g., donor cells engineered for iron resistance via FTH1 overexpression or NCOA4 knockdown) to achieve durable benefit.

Long-term immunological safety. It is critical to determine the consequences of microglial replacement for CNS-specific immune functions beyond seizure control, specifically regarding tumor immunosurveillance, the clearance of protein aggregates involved in neurodegeneration, the regulation of adaptive immune cell entry, and the maintenance of myelin integrity.

## 5 Conclusion

The rate of drug resistance in epilepsy has remained unchanged despite three decades of ASM development, suggesting that the epileptogenic substrate extends beyond neuronal excitability. The Senescent Niche Hypothesis proposes that the epileptogenic focus in DRE harbors dysfunctional senescent microglia maintained by the Iron–Senescence Axis—a mechanistic pathway linking BBB disruption, parenchymal iron deposition, and sub-lethal ferroptotic stress to irreversible microglial senescence and SASP-mediated perpetuation of the epileptogenic network (**Figure 1**).

This framework motivates a therapeutic shift from seizure suppression to immune niche reconstitution. Senolytic therapy (D+Q) and microglial replacement (MISTER), particularly when combined in a sequential protocol, offer complementary approaches to clearing and replacing the senescent niche (**Figure 2**). The convergence of non-genotoxic conditioning and CSF1R-inhibitor resistant donor cells has substantially improved the clinical tractability of microglial replacement.

However, significant challenges remain: the Iron–Senescence Axis requires direct experimental validation, MISTER has not been tested in epilepsy models, and the long-term safety and durability of microglial replacement are unknown. Addressing these questions will require iron chelation studies in TLE models, MISTER proof-of-concept experiments in epilepsy, and the development of non-invasive biomarkers for identifying and monitoring the senescent niche in patients. If validated, this approach would represent a shift from symptomatic seizure suppression to disease modification through neuroimmune niche restoration in drug-resistant epilepsy.

## Data Availability

The original contributions presented in the study are included in the article/**Supplementary Material**. Further inquiries can be directed to the corresponding author.
