# Supplementary material for: The senescent niche hypothesis: microglial dysfunction and replacement strategies in drug-resistant epilepsy
Source: Front Immunol. 2026 Apr 10;17:1807871. doi: 10.3389/fimmu.2026.1807871 (PMC13106091; doi:10.3389/fimmu.2026.1807871)
Supplement: Supplementary file 1 [file Table1.docx]

Supplementary Material

**Supplementary Table S1.** Molecular Markers of Microglial Senescence in the Context of Drug-Resistant Epilepsy and the Iron–Senescence Axis

| **Marker** | **Detection Method** | **Tissue Context** | **Functional Significance** | **Key References** |
| --- | --- | --- | --- | --- |
| ***Cell-Cycle Arrest*** | | | | |
| p16ᴵᴺᴷ⁴ᵃ (CDKN2A) | IHC, WB, RT-qPCR | Human TLE, mouse pilocarpine SE, human FCDII | CDK4/6 inhibition; enforces G1 arrest | [1, 2] |
| p21ᶜᴵᵖ¹ (CDKN1A) | IHC, WB, Patch-seq | Human TLE, human FCD, mouse pilocarpine SE | CDK2 inhibition; p53-dependent arrest | [1, 3] |
| p53 | WB, IHC | Human FCDII, mouse Mtorˢ²²¹⁵ᶠ | DDR effector; transcriptional activator of p21 | [2, 3] |
| ***DNA Damage Response*** | | | | |
| γH2AX foci | IF, Patch-seq | Human DRE (FCD, TLE) | Marker of DNA double-strand breaks | [3] |
| 53BP1 | IF | Neurodegenerative tissue (extrapolated) | DDR mediator; recruited to DSB sites | [4] |
| ATM/ATR phosphorylation | WB | In vitro senescence models | Upstream DDR kinases activating p53/p21 | [5] |
| 8-oxoguanine | IHC, ELISA | Iron-overloaded tissue | Oxidative DNA base lesion from ROS | [5] |
| ***Nuclear Envelope*** | | | | |
| Lamin B1 reduction | WB, IF, Patch-seq | Human DRE (FCD), neurodegenerative tissue | Nuclear envelope deterioration; consensus senescence marker | [3, 4] |
| ***Lysosomal / Metabolic*** | | | | |
| SA-β-gal activity | Histochemical (X-gal, pH 6.0) | Human TLE, mouse pilocarpine SE, human FCDII, mouse Mtorˢ²²¹⁵ᶠ | Expanded dysfunctional lysosomes; most widely used senescence marker | [1-4] |
| Lipofuscin accumulation | Autofluorescence, Sudan Black B | Aged brain, neurodegenerative tissue | Non-degradable lysosomal aggregates | [4] |
| Enlarged lysosomes | EM, LAMP1 IF | In vitro iron-overloaded microglia | Impaired degradative capacity | [6, 7] |
| ***Iron-Related*** | | | | |
| FTL accumulation | IHC, WB | Human AD, LBD, LATE; predicted in TLE-HS | Iron-storage subunit; marker of iron-loaded dystrophic microglia | [8] |
| FTH1 upregulation | WB | In vitro senescent cells | Ferritin heavy chain; increased iron sequestration capacity | [6] |
| Reduced ferroportin (SLC40A1) | WB, qPCR | In vitro senescent cells | Decreased iron export; contributes to intracellular iron retention | [6] |
| Decreased NCOA4 protein | WB | In vitro senescent cells (doxorubicin model) | Reduced ferritinophagy; protective lysosomal iron retention | [6] |
| ***SASP Components*** | | | | |
| IL-1β, IL-6, TNF-α | ELISA, Luminex, qPCR | Human TLE, mouse pilocarpine SE, mouse Mtorˢ²²¹⁵ᶠ | Pro-convulsant cytokines; lower seizure threshold via glutamatergic/GABAergic modulation | [1, 2, 9] |
| CCL2 (MCP-1), CCL11 | Luminex, Patch-seq | Human DRE tissue | Chemokines; monocyte recruitment and paracrine senescence signaling | [3, 10] |
| MMPs (MMP-2, MMP-9) | Zymography, WB | Human TLE, rodent SE models | Extracellular matrix degradation; PNN breakdown around interneurons; BBB compromise | [11, 12] |
| ***Morphological*** | | | | |
| Dystrophic morphology (cytorrhexis, beaded processes, spheroids) | IHC (Iba1), confocal microscopy | Human AD, LBD, LATE; human TLE-HS | Loss of ramified surveillance arbors; now recognized as morphological signature of senescence | [8, 13-16] |
| ***Surface / Functional*** | | | | |
| Loss of P2RY12 | IHC, flow cytometry | Human TLE-HS | Impaired purinergic surveillance; inability to detect neuronal distress signals | [16, 17] |
| Loss of TMEM119 | IHC | Neurodegenerative tissue (extrapolated) | Loss of homeostatic microglial identity marker | [18] |
| Upregulation of CD68, MHCII | IHC, flow cytometry | Human TLE-HS | Phagocytic/antigen-presenting shift; loss of homeostatic phenotype | [16] |

**Abbreviations:** AD, Alzheimer’s disease; DDR, DNA damage response; DSB, double-strand break; EM, electron microscopy; FCD, focal cortical dysplasia; FCDII, focal cortical dysplasia type II; FTH1, ferritin heavy chain 1; FTL, ferritin light chain; IF, immunofluorescence; IHC, immunohistochemistry; LBD, Lewy body disease; LATE, limbic-predominant age-related TDP-43 encephalopathy; MMP, matrix metalloproteinase; PNN, perineuronal net; qPCR, quantitative polymerase chain reaction; ROS, reactive oxygen species; SA-β-gal, senescence-associated β-galactosidase; SASP, senescence-associated secretory phenotype; SE, status epilepticus; TLE, temporal lobe epilepsy; TLE-HS, TLE with hippocampal sclerosis; WB, Western blot.

**Note:** Reference numbers correspond to the main manuscript bibliography. Markers are grouped by functional category. Tissue contexts marked “extrapolated” indicate that evidence derives from neurodegenerative rather than epileptic tissue, with mechanistic inference to the DRE microenvironment as discussed in the main text (Section 2.2).

1. Khan, T., et al., *Senescent Cell Clearance Ameliorates Temporal Lobe Epilepsy and Associated Spatial Memory Deficits in Mice.* Annals of Neurology, 2025.

2. Ribierre, T.o., et al., *Targeting pathological cells with senolytic drugs reduces seizures in neurodevelopmental mTOR-related epilepsy.* Nature Neuroscience, 2024. **27**(6): p. 1125–1136.

3. Ge, Q., et al., *Multimodal single-cell analyses reveal molecular markers of neuronal senescence in human drug-resistant epilepsy.* Journal of Clinical Investigation, 2025. **135**(5).

4. Gorgoulis, V., et al., *Cellular Senescence: Defining a Path Forward.* Cell, 2019. **179**(4): p. 813–827.

5. Ou, H.-L. and B. Schumacher, *DNA damage responses and p53 in the aging process.* Blood, 2018. **131**(5): p. 488–495.

6. Feng, Y., et al., *Iron retardation in lysosome protects senescent cells from ferroptosis.* Aging, 2024.

7. Lee, B.Y., et al., *Senescence‐associated β‐galactosidase is lysosomal β‐galactosidase.* Aging Cell, 2006. **5**(2): p. 187–195.

8. Shahidehpour, R.K., et al., *Dystrophic microglia are associated with neurodegenerative disease and not healthy aging in the human brain.* Neurobiology of Aging, 2021. **99**: p. 19–27.

9. Vezzani, A., et al., *The role of inflammation in epilepsy.* Nature Reviews Neurology, 2011. **7**(1): p. 31–40.

10. Acosta, J.C., et al., *A complex secretory program orchestrated by the inflammasome controls paracrine senescence.* Nature Cell Biology, 2013. **15**(8): p. 978–990.

11. Rankin‐Gee, E.K., et al., *Perineuronal net degradation in epilepsy.* Epilepsia, 2015. **56**(7): p. 1124–1133.

12. Varatharaj, A. and I. Galea, *The blood-brain barrier in systemic inflammation.* Brain, Behavior, and Immunity, 2017. **60**: p. 1–12.

13. Streit, W.J., et al., *Dystrophic microglia in the aging human brain.* Glia, 2004. **45**(2): p. 208–212.

14. Streit, W.J., et al., *Dystrophic (senescent) rather than activated microglial cells are associated with tau pathology and likely precede neurodegeneration in Alzheimerâ€™s disease.* Acta Neuropathologica, 2009. **118**(4): p. 475–485.

15. Streit, W.J., H. Khoshbouei, and I. Bechmann, *Dystrophic microglia in lateâ€onset Alzheimer's disease.* Glia, 2020. **68**(4): p. 845–854.

16. Morin-Brureau, M.l., et al., *Microglial phenotypes in the human epileptic temporal lobe.* Brain, 2018. **141**(12): p. 3343–3360.

17. Badimon, A., et al., *Negative feedback control of neuronal activity by microglia.* Nature, 2020. **586**(7829): p. 417–423.

18. Bennett, F.C., et al., *A Combination of Ontogeny and CNS Environment Establishes Microglial Identity.* Neuron, 2018. **98**(6): p. 1170–1183.e8.
